# *Metarhizium mendonceae* sp. nov.: An important biological control agent for insect pests

**DOI:** 10.1371/journal.pone.0310548

**Published:** 2025-02-10

**Authors:** Jayara Dayany da Costa Silva, Paulo Roberto Ramalho Silva, Solange Maria de França, Ibrahim A. Saleh, Francisco de Alcântara Neto, Mohamed A. El-Tayeb, Kedma da Silva Matos, Mostafa A. Abdel-Maksoud, Sarah da Silva Costa Guimarães, Alan Mario Zuffo, Gilvan Ferreira da Silva, Antônio Roberto Gomes de Farias, Marcus Eugênio Oliveira Briozo, Hamada AbdElgawad, Maruzanete Pereira de Melo

**Affiliations:** 1 Department of Agronomy, Federal Institute of Piauí, Oeiras, Piauí, Brazil; 2 Department of Plant Science, Federal University of Piauí, Teresina, Piauí, Brazil; 3 Agronomy Department, State University of Maranhão, Balsas, Maranhão, Brazil; 4 Faculty of Science, Zarqa University, Zarqa, Jordan; 5 Department of Botany and Microbiology, College of Science, King Saud University, Riyadh, Saudi Arabia; 6 Department of Agronomy Federal University of Roraima, Boa Vista, Roraima, Brazil; 7 Department of Phytopathology, Federal University of Lavras, Lavras, Minas Gerais, Brazil; 8 Embrapa Amazônia Ocidental, Manaus, Amazonas, Brazil; 9 Center of Excellence in Fungal Research, Mae Fah Luang University, Chiang Rai, Thailand; 10 Integrated Molecular Plant Physiology Research, Department of Biology, University of Antwerp, Antwerp, Belgium; 11 Department of Agricultural Planning, Federal University of Piauí, Teresina, Piaui, Brazil; Periyar University, INDIA

## Abstract

The *Metarhizium anisopliae* complex consists of 34 formally described phylogenetic species. *Metarhizium anisopliae* sensu lato has been used for decades in Brazil as a biological control agent for controlling spittlebugs in sugarcane plantations. We investigated the identities of the *Metarhizium* isolates used in mycoinsecticide formulations through multilocus phylogenetic analyses and morphological characterization for species delimitation. A well-supported clade containing only isolates from this study formed a sister group with species of *M*. *anisopliae* sensu stricto, which we described as a new taxon, *M*. *mendonceae* sp. nov. Isolates URM 8144 and URM 8145 are used in the formulation of various brands of biological insecticides; however, they have always been referred to as *M*. *anisopliae*. According to the antibiosis assay, all the isolates of this new species were able to colonize and kill *Mahanarva spectabilis* nymphs. Therefore, *M*. *mendonceae* has been used in the formulation of mycoinsecticides for several decades under the name *M*. *anisopliae*.

## Introduction

The genus *Metarhizium* encompasses entomopathogenic fungi that infect a wide range of arthropod orders, leading to green muscardine disease. *Metarhizium* spp. are cosmopolitan, occurring in arthropods (causing infections), in the soil, and in the rhizosphere, as well as within plants as endophytes [1, 2]. These fungi are of considerable interest due to their potential in controlling agricultural pests and disease vectors. Additionally, they can function as plant growth promoters and play a role in nutrient cycling [3, 4].

*Metarhizium anisopliae* (Metschn.) Sorokin (the type species of the genus) was originally described in Russia as a pathogen of *Anisoplia austriaca* (*Coleoptera*) (Herbst, 1783). This fungus has been reported to infect over 200 insect species, which are widely distributed in both tropical and temperate regions [5]. The genus *Metarhizium* consists of generalist microorganisms with a global distribution. Many *Metarhizium* species are now classified within the *M*. *anisopliae* or *M*. *flavoviride* species complex, with some species exhibiting host-specific adaptations [6].

This ability to infect a variety of arthropod species has been explored as an alternative strategy for the biological control of insect pests and disease vectors [7]. *Metarhizium anisopliae* has been utilized to manage insect pests in numerous countries, including Brazil, Iran, Japan, Thailand, and the USA [8–10]. In Brazil, in 1969, Arthur P. Mendonça and Edimilson Jacinto Marques observed that under natural conditions, *M*. *anisopliae* colonized *Mahanarva fimbriolata*. Following this observation, the authors began to multiply and apply the fungus as a biological insecticide. Consequently, biological control using *M*. *anisopliae* has proven to be an effective method for combating spittlebugs in sugarcane plantations [11]. Currently, mycoinsecticides produced with *M*. *anisopliae* are estimated to be applied to over two million hectares of sugarcane plantations to reduce the populations of *M*. *fimbriolata* and *M*. *spectabilis* [12, 13]. In sugarcane production areas in northeastern Brazil, *Metarhizium anisopliae* (Metsch.) Sorok. has been used with significant success to control the leafhopper, representing one of the most successful biological control programs in Latin America [14].

Prior to the 2000s, the taxonomy of *Metharizium* was based on morphological characteristics. However, the taxonomy of *Metarhizium* spp. has been challenging due to the significant morphological similarities among species, particularly in features such as conidiogenous cells and conidial dimensions and shape [5, 15]. The first DNA sequencing studies were conducted by Driver and Collaborators, who sequenced the ITS genomic region and identified four phylogenetic lineages of *Metarhizium*: *Metarhizium anisopliae* var. *acridum*, *M*. *anisopliae* var. *anisopliae*, *M*. *anisopliae* var. *lipidiotae*, and *M*. *anisopliae* var. *maju* [16]. Subsequently, these varieties, along with *M*. *guizhouense*, *M*. *pingshaense*, and *M*. *tai*, were elevated to species status based on multigene phylogenetic analysis [5]. With the advancement of molecular biology tools, the taxonomy of several fungal genera has been clarified, demonstrating that the phylogenetic species concept is the most appropriate to apply. For instance, research conducted by Mongkolsamrit and collaborators was cited, where it was confirmed that *M*. *carneum* was relocated to the *Keithomyces* clade, *M*. *khaoyaiense* to *Purpureomyces*, and *M*. *kusanagiense* to *Yosiokobayasia* [17].

The *M*. *anisopliae* complex consists of 21 formally described species, among which *Metarhizium pingshaense*, *M*. *anisopliae* s.str., *Metarhizium robertsii*, *Metarhizium brunneum*, and *Metharizium humberi* were assigned to the PARB clade. The other species (*M*. *majus*, *M*. *indigoticum*, and *M*. *guizhouense*) were assigned to the MGT clade [5]. The other species of the *M*. *anisopliae* complex do not belong to any of these clades; they are represented by *Metarhizium acridum*, *Metarhizium lepidiotae*, *Metarhizium globosum*, and *Metarhizium kalasinense* [18]; *Metahrizium alvesii*, *Metarhizium baoshanense*, *Metarhizium phasmatodeae*, *Metarhizium clavatum*, *Metarhizium sulphureum*, *Metarhizium gryllidicola*, and *Metarhizium brittebankisoides* [6, 18–20].

In biological control, mainly with the use of fungi-based microorganisms, it is fundamentally important to identify the biological agents of agricultural pests and understand their host range. The biodiversity of entomopathogenic fungi in tropical ecosystems has been poorly investigated. In addition, research involving the characterization of *Metarhizium* spp. using the phylogenetic concept of species identification is scarce in Brazil. This study aimed to identify *Metarhizium* isolates using multilocus phylogenetic analyses (*TEF1-α*, *Β-TUB*, *RPB1*, and *RPB2)* to define the phylogenetic position of the isolates within the *M*. *anisopliae* complex and verify their potential as biocontrol agents for *M*. *spectabilis*. *Metarhizium mendonceae* sp. nov. was introduced based on morpho-molecular evidence.

## Material and methods

### Isolate collection

*Metarhizium* isolates were collected from mummified insects, spittlebugs, and fruit flies. Two isolates were obtained from the "Oldemar Cardim Abreu" Entomopathogenic Microorganism Collection at the Experimental Center (Laboratory of Biological Control) of the São Paulo Biological Institute (IBCB). Two other isolates were provided by Fitoagro—Controle Biológico (São Miguel dos Campos). Single isolates obtained by the single-spore technique were deposited in the fungal culture collection (Micoteca—URM) of the Federal University of Pernambuco, Recife, Brazil (Table 1). The ex-type isolate was deposited in the microbial collection of Embrapa (Embrapa Cernargem) and in the fungal culture collection of UFPE plants.

**Table 1:** The *metarhizium* isolates used in this study and their culture collection, substrate, location, and GenBank accession information.

Species	Culture collection number(s)^a^	Host/substrate	Origin	GenBank accession numbers^b^			
				*TEF-1α*	*β-TUB*	*RBP1*	*RBP2*
*M*. *mendoncearum*	URM 8139	Unknown	Brazil	MZ394804	MZ394810	MW885971	MZ394798
*M*. *mendoncearum*	URM 8140	*M*. *posticata*	Brazil	MZ394805	MZ394811	MW885972	MZ394799
*M*. *mendoncearum*	URM 8141	*M*. *posticata*	Brasil	MZ304806	MZ394812	MW885973	MZ394800
*M*. *mendoncearum*	URM 8142	*M*. *posticata*	Brazil	MZ394807	MZ394813	MW885974	MZ394801
*M*. *mendoncearum*	**URM 8143T**	***A*.*alveata***	**Brazil**	**MZ394808**	**MZ394814**	**MW885975**	**MZ394802**
*M*. *mendoncearum*	URM 8144	*M*. *fimbriolata*	Brazil	MZ394809	MZ394813	MW885976	MZ394803
*M*. *acridum*	ARSEF 7486T	Orthoptera	Niger	EU248845	EU248813	EU248897	EU248925
*M*. *acridum*	ARSEF 324	Orthoptera	Australia	EU248844	EU248812	EU248916	EU248944
*M*. *alvesii*	CG1123T	Soil	Brazil	KY007614	KY007611	KY007612	KY007613
*M*. *anisopliae*	ARSEF 7487T	Orthoptera	Eritrea	DQ463996	EU248822	DQ468355	DQ468370
*M*. *anisopliae*	ARSEF 7450	Coleoptera	Australia	EU248852	EU248823	EU248904	EU248932
*M*.*brunneum*	ARSEF 2107T	Coleoptera	USA	EU248855	EU248826	EU248907	EU248935
*M*. *brunneum*	ARSEF 4179	Soil	Australia	EU248854	EU248825	EU248906	EU248934
*M*. *frigidum*	ARSEF 4124T	Coleoptera	Australia	DQ464002	EU248828	DQ468361	DQ468376
*M*. *globosum*	ARSEF 2596T	Lepidoptera	India	EU248846	EU248814	EU248898	EU248926
*M*. *guizhouense*	CBS 258.90 T	Lepidoptera	China	EU248862	EU248834	EU248914	EU248942
*M*. *guizhouense*	ARSEF 6238	Lepidoptera	China	EU248857	EU248830	EU248909	EU248937
*M*. *humberi*	IP 46T	Soil	Brazil	JQ061205	MH837547	MH837556	MH837565
*M*. *humberi*	IP 151	Soil	Brazil	JQ061208	MH837552	MH837561	MH837570
*M*. *indigoticum*	NBRC 100684 T	Lepidoptera	Japan	KJ398784	KJ398544	KJ398544	KJ398692
*M*. *lepidiotae*	ARSEF 7488T	Coleoptera	Australia	EU248865	EU248837	EU248917	EU248945
*M*. *lepidiotae*	ARSEF 7412	Coleoptera	Australia	EU248864	EU248836	EU248916	EU248944
*M*. *majus*	ARSEF 1946	Coleoptera	Philippines	EU248867	EU248839	EU248919	EU248947
*M*. *pinghaense*	CBS 257.90T	Coleoptera	China	EU248850	EU248820	EU248902	EU248930
*M*. *pinghaense*	ARSEF 4342	Coleoptera	Solomon Islands	EU248851	EU248821	EU248903	EU248931
*M*. *robertsii*	ARSEF 7501	-	Australia	EU248849	EU248818	EU248901	EU248929
*M*. *robertsii*	ARSEF 727	Orthoptera	Brazil	DQ463994	EU248816	DQ468353	DQ468368

^a^ Abbreviations for collections: ARSEF, USDA-ARS Collection of Entomopathogenic Fungal Cultures, Ithaca, NY, USA; CBS, Centraalbureau voor Schimmelcultures, Utrecht, the Netherlands; CG, Invertebrate-Associated Fungal Collection at Embrapa Genetic Resources and Biotechnology, Brasília, Brazil; IP, Institute of Tropical Pathology and Public Health, Federal University of Goiás, Goiânia, Goiás, Brazil; NBRC, National Institute of Technology and Evaluation, Biological Resource Center, Chiba, Japan; URM, “Micoteca” of the Universidade Federal de Pernambuco, Recife, Brazil.

^b^ *TEF-1α*: translation elongation factor 1α; *β-TUB*: β-tubulin; *RPB1*: largest subunit of RNA polymerase II; *RPB2*: second-largest subunit of RNA polymerase II.

T = ex-type isolate

### Morphological evaluation

The isolates were subsequently grown in 2% malt extract, after which the Petri dishes were incubated at 25°C under a 12 h photoperiod. Conidia from the isolates were transferred to culture media in Petri dishes and slides containing solidified culture media. The slides were covered with coverslips, placed in Petri dishes containing a portion of cotton moistened with distilled water (both sterilized) and incubated in a humidified chamber. Conidia from cultures grown in Petri dishes were suspended in distilled water and Tween (a wetting agent) and subsequently used in microscopic preparations. Images of conidia and hyphae were captured (software TSVIEW 6.13.2) through an optical microscope coupled with a digital camera (TUCSEN). The length and width of 20 conidia were measured for each isolate using the software MicroMeasure.3.3.0.

### DNA extraction, PCR amplification, and sequencing

Fungal cultures were grown in malt extract, and DNA was extracted using the CTAB protocol [21]. DNA concentration and quality were evaluated via a Nanodrop spectrophotometer and by electrophoresis on 0.8% agarose gels.

Four gene fragments were amplified using the following primer pairs: tef71F (5’-CAAAATGGGTAAGGAGGASAAGAC-3’)/tef 9997R (5’-CAGTACCGGCRGCRATRATSAG-3’) [22] for the *translation elongation factor 1α* (*TEF-1α*) gene; *tub2*FD (5’-GTBCACCTYCARACCGGYCARTG-3’)/*tub4*RD (5’-CCRGAYTGRCCRAARACRAAGTTGTC-3’) [5] for the *β-tubulin* (*β-TUB*) gene; *rpb1*A_f_ (5’-GARTGYCCDGGDCAYTTYGG-3’) [23] /Fa (5’-CAYAARGARTCYGATGGGWC-3’) [24] for the largest subunit of RNA polymerase II (*RPB1*); and s7cF (5’-ATGGGYAARCAAGCYATGGG-3’) [25] for the second largest subunit of RNA polymerase II (*RPB2*). These markers were amplified by PCR using a mixture (25 μl final volume) comprising approximately 150 ng of genomic DNA, 1x PCR buffer with 2.0 mM MgCl_2_, 0.4 mM dNTPs, 1 U of Taq polymerase, and 0.5 μM of each primer. Thermal cycling was standardized for all markers as 3 min at 95°C, followed by 35 cycles of 1 min at 95°C, 1 min at the appropriate annealing temperature for the primers used, 1 min at 72°C, and 10 min at 72°C. The generated amplicons were visualized on agarose gels, while their sizes were measured with a 1 kb DNA Ladder (Invitrogen).

PCR products were treated with polyethylene glycol (20% PEG) [26] and sequenced by the chain termination method (Sanger sequencing) using the BigDye^™^ Terminator v3.1 cycle sequencing kit in the 3,500 Genetic Analyzer (Applied Biosystems^™^) in standard mode.

### Phylogenetic analysis

Consensus sequences were assembled through bidirectional sequencing of DNA using the software SeqAssem v. 07/2008 (SequentiX—Digital DNA Processing). Additional sequences of *M*. *anisopliae* isolates from different hosts were obtained from GenBank (Table 1). The sequences were aligned through the MAFFT online service (https://MAFFT.cbrc.jp/alignment/server/large.html [27] using the default configurations and treated using GBLOCKS v.0.91.1 [28] with all the gap positions allowed. The software MrModeltest 2.3 [29] was used to determine the best model of nucleotide evolution based on the Akaike information criterion (AIC). K80+I for *TEF-1α*, K80+G for *Β-TUB*, and SYM+G for *RPB1* and *RPB2* were the best-fit models for the data. The concatenated four-locus alignment included 28 ingroup taxa and 2,633 characters, including gaps. The gene boundaries were identified as 1–405 bp for *Β-TUB*, 406–1,050 for *RPB1*, 1,051–1,913 for RPB2, and 1,914–2,633 for *TEF-1α*.

Bayesian phylogenetic analysis was performed via the CIPRES web portal [30] through MrBayes v.3.2 [31]. Four independent and simultaneous Markov chains were run over 3,000,000 generations, with tree sampling every 300 generations. The first 2,500 trees were discarded for burn-in. The remaining 7,500 trees were used to calculate the posterior probabilities of the branches, determined by the consensus of most of the sampled trees. The phylogenetic trees were visualized in FigTree [32] and exported to graphic software. *Metarhizium frigidium* ARSEF 4124 was used as the outgroup. All the sequences generated in this study were deposited in the NCBI GenBank database (www.ncbi.nlm.nih.gov; Table 1). Maximum likelihood phylogenetic estimation was performed using IQ-TREE v.1.6.12 [33].

### Nomenclature

In addition, new names contained in this work have been submitted to MycoBank from where they will be made available to the Global Names Index. The unique MycoBank number can be resolved and the associated information viewed through any standard web browser by appending the MycoBank number contained in this publication to the prefix http://www.mycobank.org/MB/. The online version of this work is archived and available from the following digital repositories.

### Antibiosis assay

All the strains obtained in this study were evaluated for their ability to colonize and kill *M*. *spectabilis* nymphs. Nymphs were distributed in the exposed roots of the sugarcane seedlings for the application of *M*. *mendonceae* isolates. To prepare the inoculum suspensions, the PDA culture medium containing the fungal colonies was washed with sterile distilled water and a wetting agent (Tween^®^ 80) at 0.1% v/v. The inoculum concentration was quantified with a Neubauer chamber under optical microscopy, and the concentration was adjusted to 1 × 10^9^ conidia/mL [34]. An aliquot of 1 mL of the spore suspension was inoculated onto each nymph using a spray bottle. The negative control consisted of a nymph sprayed with sterile distilled water. The experiment was performed in accordance with a completely randomized design with ten replicates (spittlebug nymphs) for each treatment (six isolates). Seedlings with spittlebug nymphs were placed inside plastic bottles (1 L), after which the plant shoot was exposed and maintained at a temperature of 29 ± 2°C, 70% relative humidity, and a 12 h photoperiod.

## Results

### Phylogeny

**Fig 1:**
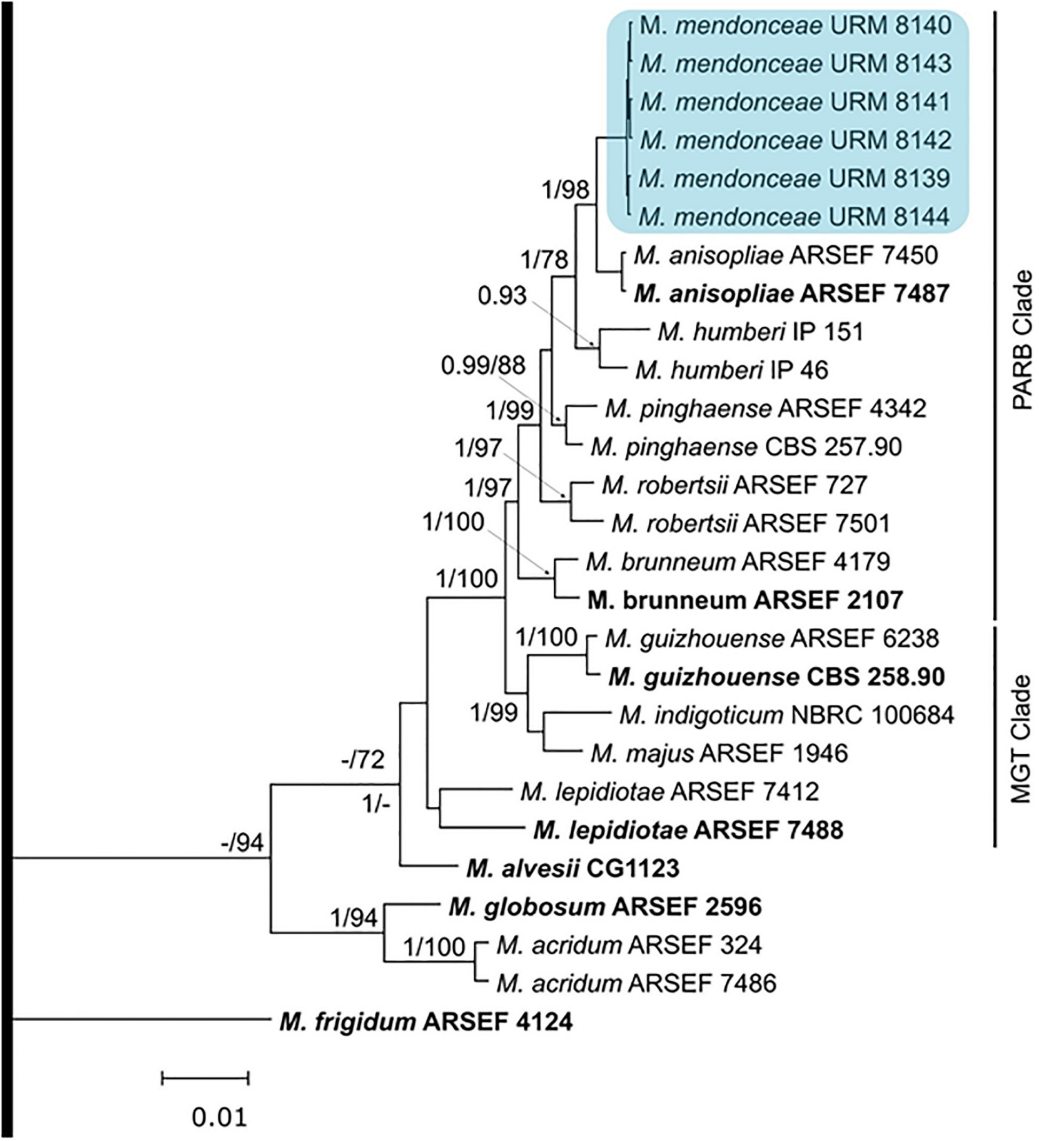
Bayesian phylogenetic tree based on concatenated *TEF1*α-Β-TUB-RPB1-RPB2 sequences showing relationships among species of the *Metarhizium anisopliae* complex. Isolates from this study from *Mahanarva spectabilis*, *Anastrepha* sp., and soil are highlighted in bold. Bayesian posterior probability (PP) values > 0.9 are indicated above the nodes. The symbol T refers to ex-type isolates. This tree is rooted in *M*. *fluorescens*.

The *TEF1-α*, *Β-TUB*, *RPB1*, and *RPB2* gene sequences of the *Metarhizium* isolates were compared with similar sequences from strains of other species that are part of the *Metarhizium anisopliae* complex using BLASTn, and it was observed that the isolates belonged to the *M*. *anisopliae* complex. According to the phylogenetic analysis of the four sequenced gene regions, the isolates under study formed a monophyletic group with bootstrap values ranging from 0.91 to 0.98%. The six isolates of *Metarhizium* (URM 8139, URM 8140, URM 8142, URM 8143, URM 8144, and URM 8145) formed a single, strongly supported clade (PP = 1) that differed from that of other species of the *M*. *anisopliae* complex in the Bayesian tree obtained from the *Β-TUB*, *TEF1-α*, *RPB1*, and *RPB2* gene sequences (S1–S4 Figs) and multilocus alignments (Fig 1). The six *Metarhizium* isolates differed from the other species in the PARB clade by 105 nucleotides in the *Β-TUB*, *TEF1-α*, *RPB1*, and *RPB2* gene regions (S1 Table). Seven nucleotides were unique to the six new isolates (S1 Table). Therefore, these isolates belong to a new species, *Metarhizium mendonceae* sp. nov., a sister of *M*. *anisopliae* sensu stricto.

### Taxonomy

**Fig 2:**
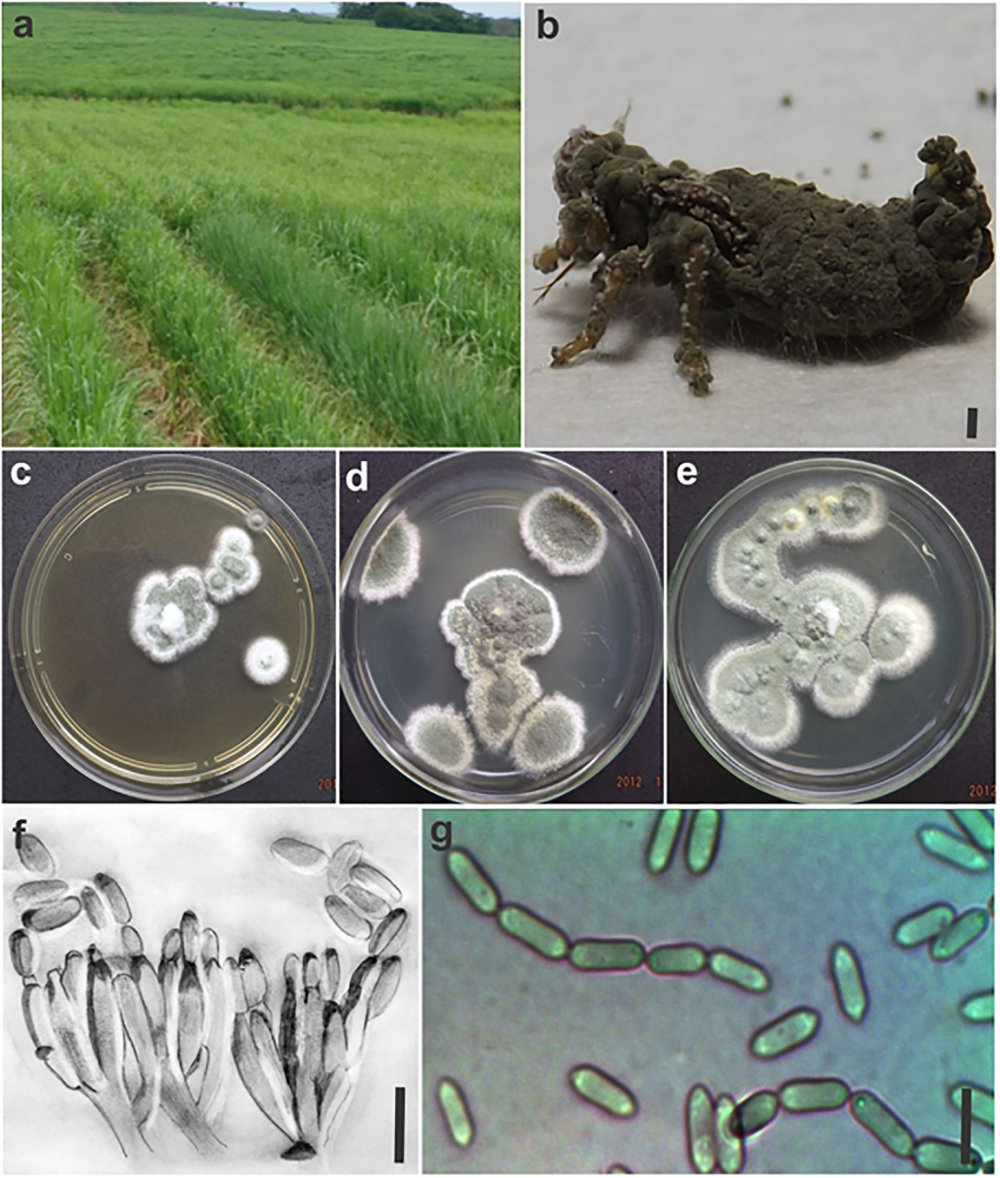
*Metarhizium mendonceae*: a) Sugarcane cultivation area where samples of the fungus were collected. Postmortem signs (fungus outgrowth) of infection in an adult of *Mahanarva spectabilis*; c-d-e) Colony grown in 2% malt extract after 15 days at 25°C and a 12-h photoperiod; f) Drawing of phialides; g) Production of conidia in chains. Scale bars = 10 μm.

*Metarhizium mendonceae* J.D.C. Silva, K.S. Matos & M.P. Melo, **sp. nov**. (Fig 2).

MycoBank: 840851

*Etymology*: In reference to the entomologist Arthur P. Mendonça, who first observed this fungal epizootic in *Mahanarva fimbriolata* in Northeast Brazil, successful biological control of spittlebugs was started in sugarcane plantations.

*Holotype*: Brazil; state of Piauí, municipality of Teresina, on a fruit farm: culture dried in 2% malt extract, isolated from mummified fruit flies, 2016, collected by Jayara Dayany da Costa Silva—VIC. 47500 *Ex-type culture*: URM 8143 and CG 1465.

*GenBank accession numbers*: *TEF1-α* = MZ394808; *Β-TUB* = MZ394814; *RPB1* = MW885975; *RPB2* = MZ394802.

*Habitat*: *Mahanarva posticata*, *Mahanarva spectabilis*, *Anastrepha alveata*, and soil.

*Description*: Colonies were grown in 2% malt extract and exhibited an increase in diameter of 30 mm/day after 15 days at 25°C under a 12-h photoperiod; the plants were first white in color and subsequently became olive green. A 15-day-old colony produced gray conidial masses with a dry appearance (Fig 2B). The conidia are unicellular and cylindrical and 3.64–6.08 × 1.29–1.82 μm in length; they are produced in chains arising from ovoid-ellipsoid phialides that are 10.91 × 2.12 μm in size and formed on branched conidiophores (Fig 2C and 2D). A sexual morph was not observed.

*Other examined specimens*: URM 8139, URM 8140, URM 8142, URM 8144, and URM 8145.

*Notes*: *Metarhizium mendonceae* cannot be distinguished from most species of the *Metarhizium anisopliae* complex solely on the basis of the evaluation of conidia, presence of conidiogenous cells, and culture characteristics; these species are members of the PARB clade along with *M*. *robertsii*, *M*. *humberi*, *M*. *pinghaense*, *M*. *brunneum*, and *M*. *anisopliae*. Although the species present different morphometries, the size of the structures is not a reliable marker, as these dimensions can change due to nutritional variations (culture medium).

*Phylogenetic differentiation*: According to the phylogenetic analyses, *Metarhizium mendonceae* has a different PARB according to the individual *TEF1-α*, *Β-TUB*, *RPB1*, and *RPB2* gene sequences and the concatenated dataset (*TEF1-α*, *Β-TUB*, *RPB1*, and *RPB2*). It is in close phylogenetic proximity to *M*. *anisopliae* sensu stricto.

### Antibiosis assay

All *M*. *mendonceae* isolates colonized and killed *M*. *spectabilis* nymphs approximately five days after inoculation, with mortality rates ranging from 20 to 70%. URM 8140, URM 8141, and URM 8145 were highly virulent (mortality rate of 70%), while the isolate URM 8143 had lower virulence.

## Discussion

This paper reports a new phylogenetic species, *M*. *mendonceae* sp. nov., in the PARB clade of the *M*. *anisopliae* complex consisting of six phylogenetically distinct species.

*Metarhizium mendonceae* presents cylindrical phialides with the formation of a compact hymenium, and its conidia are ovoid to cylindrical. Several species within the *Metarhizium* complex exhibit these morphological markers, including *M*. *anisopliae* s. strictu. Due to these similar morphological characteristics, mycologists have faced significant challenges in unraveling the taxonomy of this genus for over 100 years. The delimitation of these species is strongly enhanced by the uniqueness premium under the Genealogical Concordance Phylogenetic Species Recognition (GCPSR) [35], where reciprocal monophyly showed strong Bayesian posterior probability from combined sequence analyses of *TEF-1α*, *β-TUB*, *RBP1*, and *RBP2*. Using multilocus phylogeny, the isolates in this study formed a sister clade to *M*. *anisopliae*, a lineage called *M*. *mendonceae*. The use of molecular biology tools through multilocus DNA sequencing is the most efficient strategy for determining the taxonomy of the genus *Metarhizium*. In a study of the identification and diversity of *Metarhizium* species, the use of DNA sequencing made the method more efficient and effective [36].

Many species of the *M*. *anisopliae* complex (mostly from tropical regions) have been described in recent years based on multilocus phylogeny [20, 37]. Three *Metarhizium* species belonging to the *M*. *anisopliae* complex were recently described in Brazil. *Metarhizium humberi*, *M*. *alvesii*, and *M*. *robertsii* were described to honor Brazilian researchers who significantly contributed to the biological control of insect pests (*e*.*g*., *M*. *alvesii* was described in honor of Sergio Batista Alves, a researcher at the Luiz de Queiroz College of Agriculture—ESALQ).

This research demystifies the identification of an important biological agent for pest insects, as the active ingredient was mistakenly used for many years as *M*. *anisopliae*. This result demonstrates that naming *Metarhizium* species only through morphological markers is obsolete and that the correct identification of biological control agents is extremely important since each species has different levels of virulence, pathogenic patterns, and adaptation to different ecosystems [38]. In Brazil, many studies on the prospecting of *Metarhizium* isolates have been carried out using only morphology. Therefore, some studies have demonstrated different levels of virulence in the isolates, revealing that there may be more than one taxon within this collection of isolates.

The distribution of *M*. *mendonceae* is unknown due to the small number of collected samples. However, it is believed that this species is widely distributed in Brazil due to its use as an active ingredient of biological insecticides in sugarcane production areas in the Northeast, Southeast, and Central-west regions of Brazil. Although *Metarhizium mendonceae* has been used in the production of biological products since the 1970s, under the name *M*. *anisopliae*, its association with beneficial insects such as pollinators, predators, and parasitoids is not known. For instance, in this research, the tested isolates were obtained from *M*. *posticata* and *A*. *alveata*. Additional evidence that reinforces this hypothesis of the wide distribution of *M*. *mendonceae* in Brazil is the dispersal capacity of the *Metarhizium* species of the PARB clade, as the conidia are small and can be transported by air currents. In addition, insects with latent infection can potentiate the spread of spores [36]. Several studies have shown that the species *M*. *anisopliae*, *M*. *robestsii*, and *M*. *humberi* are widely distributed in several Brazilian biomes and are frequently obtained from soils [37–40]. The described species of the PARB clade are widely distributed on the American, Asian, and African continents.

In the current systematics for entomopathogenic fungi such as *Metarhizium*, the application of the morphological species concept is not sufficient for clear species delimitation [5]. Few species in the *M*. *anisopliae* complex have morphological descriptors that can be used as differential characters for identification at the species level. For example, *M*. *globosum* has globose conidia and can be morphologically differentiated from the other species of the complex [5]. Other species, such as *M*. *cicadae* and *M*. *cylindrosporum*, have long conidia. However, the identification process may be flawed even when using a differential morphological marker, leading to misidentification [5, 6].

Sugarcane producers and biological control companies in Brazil use *M*. *anisopliae* sensu lato as an active ingredient to formulate biological insecticides. This study showed that several isolates of this complex belong to a new species named *M*. *mendonceae*. The isolates URM 8144 and URM 8145 have been used as active ingredients in various biological insecticides for decades, while the isolate URM 8145 is used to formulate biological products by companies such as Bioenergia do Brasil, JCO LTDA, Proibio LTDA, Biocontrol LTDA, Fioagro Controle Biológico, and Agrivalle LTDA. Produtos comerciais These products are used for the biological control of several pests, such as *M*. *fimbriolata*, *Notozulia entreriana*, and *Deois flavopita* [41].

Although *M*. *mendonceae* (URM 8144 and URM 8145) is an active ingredient in the formulation of biological products targeting insects of the order Hemiptera (such as *M*. *fimbriolata*, *Notozulia entreriana*, and *Deois flavopicta*), this species of fungus could be tested against other orders of agriculturally important insects. In Brazil, genetic resistance to some chemical insecticides has been observed in certain pests, prompting future studies to verify the efficiency of this fungus in controlling key pests, such as defoliating caterpillars in soybean and corn crops. It is believed that *M*. *mendonceae* can be used in integrated pest management, especially for stink bugs, which are very common in Brazilian plantations. These pests exhibit low susceptibility to fungal infection, likely due to the composition of their cuticle layer [42]. Research has shown that a strategy to overcome this chemical barrier includes using sublethal doses of chemical insecticides in combination with entomopathogenic fungi. For instance, the application of *M*. *anisopliae* (strain CG 168) amended with a sublethal dose of thiamethoxam significantly increased the mortality of the rice stalk borer (*Tibraca limbativentris*) [43].

It is believed that the use of *M*. *mendonceae* in the control of insect vectors and diseases in humans could be a viable alternative. However, research would be necessary to confirm this application. For example, *M*. *humberi* has been used in the biological control of *Aedes aegypti* and *Anopheles gambiae*, which are vectors of Chagas disease [44–48].

There is no information available on the survival ability of *M*. *mendonceae*; however, it is believed that this species survives either by decomposing organic residues or through the formation of chlamydospores. Species of the *M*. *anisopliae* complex present a survival strategy that involves the ability to decompose organic matter, colonize different arthropod species, and multiply in the rhizosphere of different plants. Furthermore, *M*. *anisopliae*, *M*. *humberi*, and *M*. *robertsii* produce microsclerotia, which are important structures for maintaining the viability of these species under adverse conditions [49]. Previous studies have shown that the main species present in Brazilian soils, *M*. *robertisii* and *M*. *brunneum*, are generalists and can colonize roots and several insects [20, 37, 38, 40] characterized several isolates collected from Brazilian soils and concluded that all of them were *M*. *humberi*, a new species of the *M*. *anisopliae* complex. On the other hand, in a survey carried out in different locations in the state of Goiás, Brazil, only one isolate (out of 63) was identified as *M*. *anisopliae* sensu stricto [40]. This information shows that *M*. *anisopliae* sensu stricto is uncommon in Brazil.

Based on the literature, it is believed that a wide diversity of *Metarhizium* species is found in Brazil due to the ability of these fungi to colonize diverse arthropods in different biomes, combined with the tropical conditions and environmental factors that favor the biological development and survival of *Metarhizium* spp. It is necessary to obtain several isolates from different substrates and identify them using the phylogenetic species concept to prove this hypothesis. In this way, the present study introduces *M*. *mendonceae* sp. nov., justified by both morphological and molecular evidence. This fungus is an active ingredient of an important mycoinsecticide commercialized in Brazil and is used for the biological control of pests in several crops. The correct identification of this species could be useful for future genetic research and for possible studies investigating the identification of molecules with enzymatic potential.

## Supporting information

S1 Fig Fig 3. Bayesian phylogenetic tree based on *Β-TUB* sequences showing relationships among species of the *Metarhizium anisopliae* complex. Isolates from this study from *Mahanarva spectabilis*, *Anastrepha* sp., and soil are highlighted in bold. Bayesian posterior probability (PP) values > 0.7 are indicated above the nodes. The symbol T refers to ex-type isolates. This tree is rooted in *M*. *fluorescens*.
(JPG)

S2 Fig Fig 4. Bayesian phylogenetic tree based on *TEF1α* sequences showing relationships among species of the *Metarhizium anisopliae* complex. Isolates from this study from *Mahanarva spectabilis*, *Anastrepha* sp., and soil are highlighted in bold. Bayesian posterior probability (PP) values > 0.7 are indicated above the nodes. The symbol T refers to ex-type isolates. This tree is rooted in *M*. *fluorescens*.
(JPG)

S3 Fig Fig 5. Bayesian phylogenetic tree based on *RPB1* sequences showing relationships among species of the *Metarhizium anisopliae* complex. Isolates from this study from *Mahanarva spectabilis*, *Anastrepha* sp., and soil are highlighted in bold. Bayesian posterior probability (PP) values > 0.7 are indicated above the nodes. The symbol T refers to ex-type isolates. This tree is rooted in *M*. *fluorescens*.
(JPG)

S4 Fig Fig 6. Bayesian phylogenetic tree based on *RPB2* sequences showing relationships among species of the *Metarhizium anisopliae* complex. Isolates from this study from *Mahanarva spectabilis*, *Anastrepha* sp., and soil are highlighted in bold. Bayesian posterior probability (PP) values > 0.7 are indicated above the nodes. The symbol T refers to ex-type isolates. This tree is rooted in *M*. *fluorescens*.
(JPG)

S1 Table Table 2. Summary of polymorphic sites found within sequences of the *Β-TUB*, *TEF1-α*, *RPB1*, and *RPB2* gene regions for all known *Metarhizium* species in the PARB clade, including *Metarhizium mendonceae*, which was described in this study. Polymorphic nucleotides unique to *M*. *mendonceae* are highlighted in bold.
(XLSX)
