# Supplementary figures and images for: Metarhizium mendonceae sp. nov.: An important biological control agent for insect pests
Source: PLoS One. 2025 Feb 10;20(2):e0310548. doi: 10.1371/journal.pone.0310548 (PMC11809916; doi:10.1371/journal.pone.0310548)

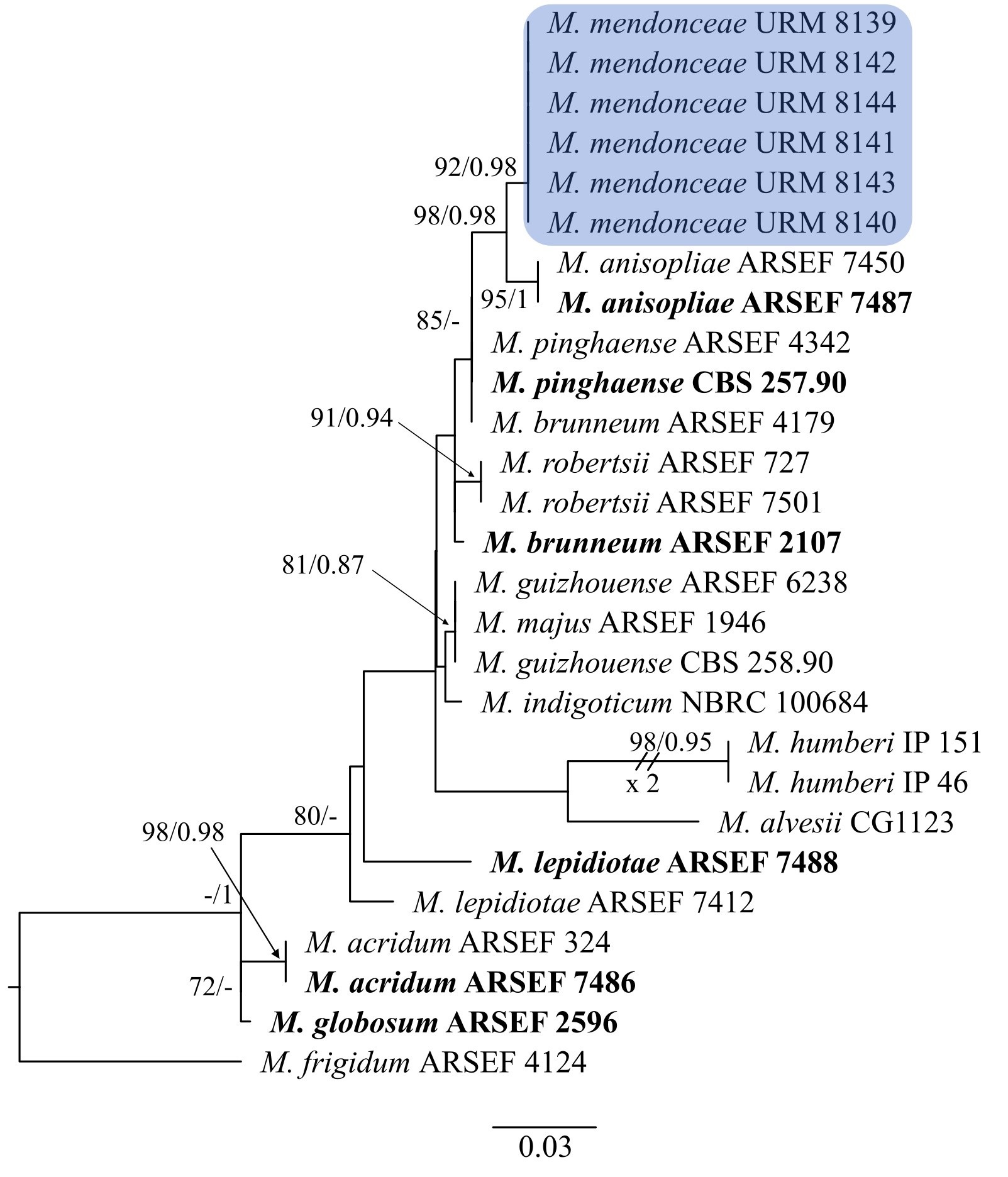

Supplement: S1 Fig — Bayesian phylogenetic tree based on Β-TUB sequences showing relationships among species of the Metarhizium anisopliae complex. Isolates from this study from Mahanarva spectabilis, Anastrepha sp., and soil are highlighted in bold. Bayesian posterior probability (PP) values > 0.7 are indicated above the nodes. The symbol T refers to ex-type isolates. This tree is rooted in M. fluorescens. (JPG) [file pone.0310548.s001.jpg]

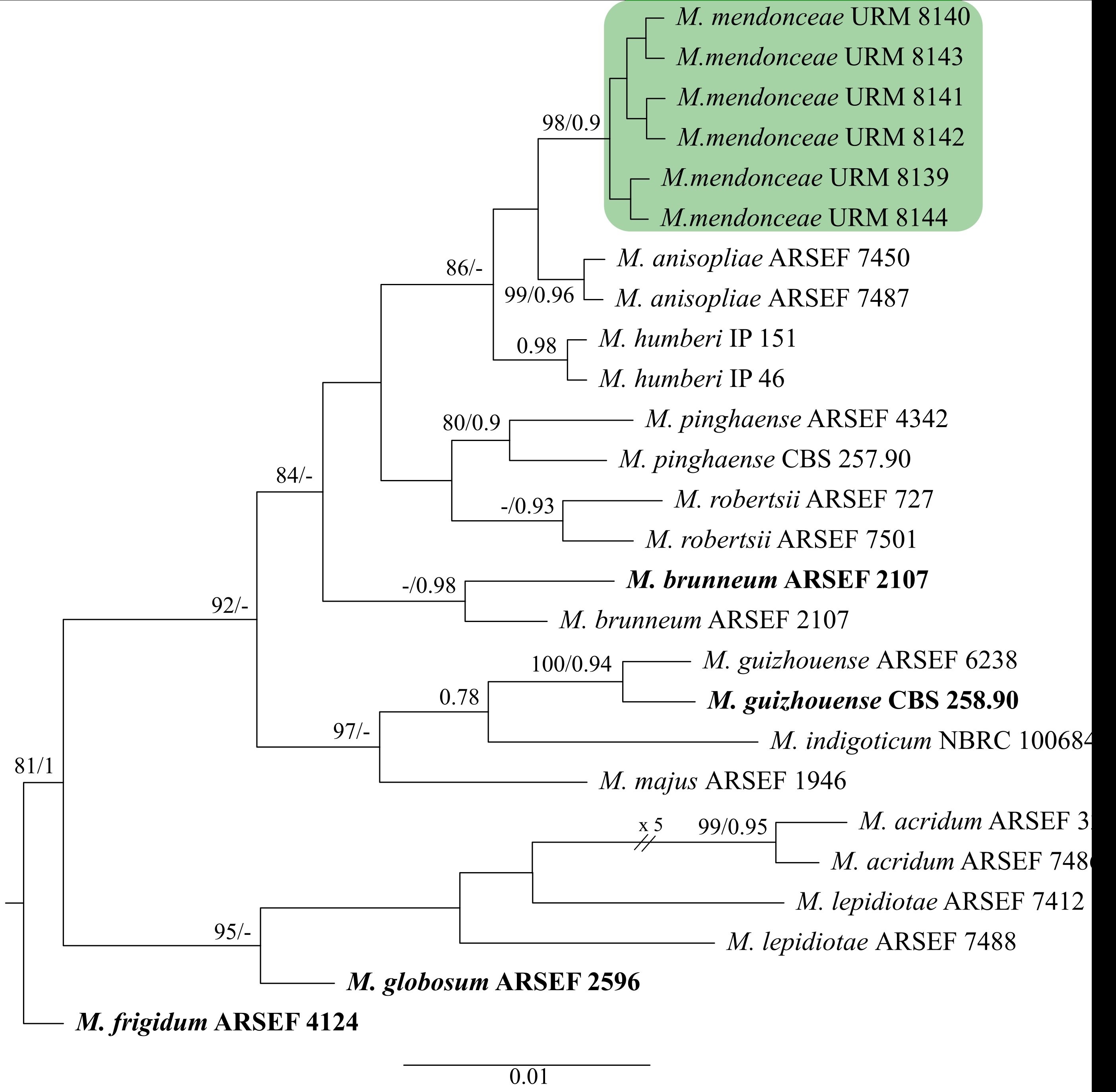

Supplement: S2 Fig — Bayesian phylogenetic tree based on TEF1α sequences showing relationships among species of the Metarhizium anisopliae complex. Isolates from this study from Mahanarva spectabilis, Anastrepha sp., and soil are highlighted in bold. Bayesian posterior probability (PP) values > 0.7 are indicated above the nodes. The symbol T refers to ex-type isolates. This tree is rooted in M. fluorescens. (JPG) [file pone.0310548.s002.jpg]

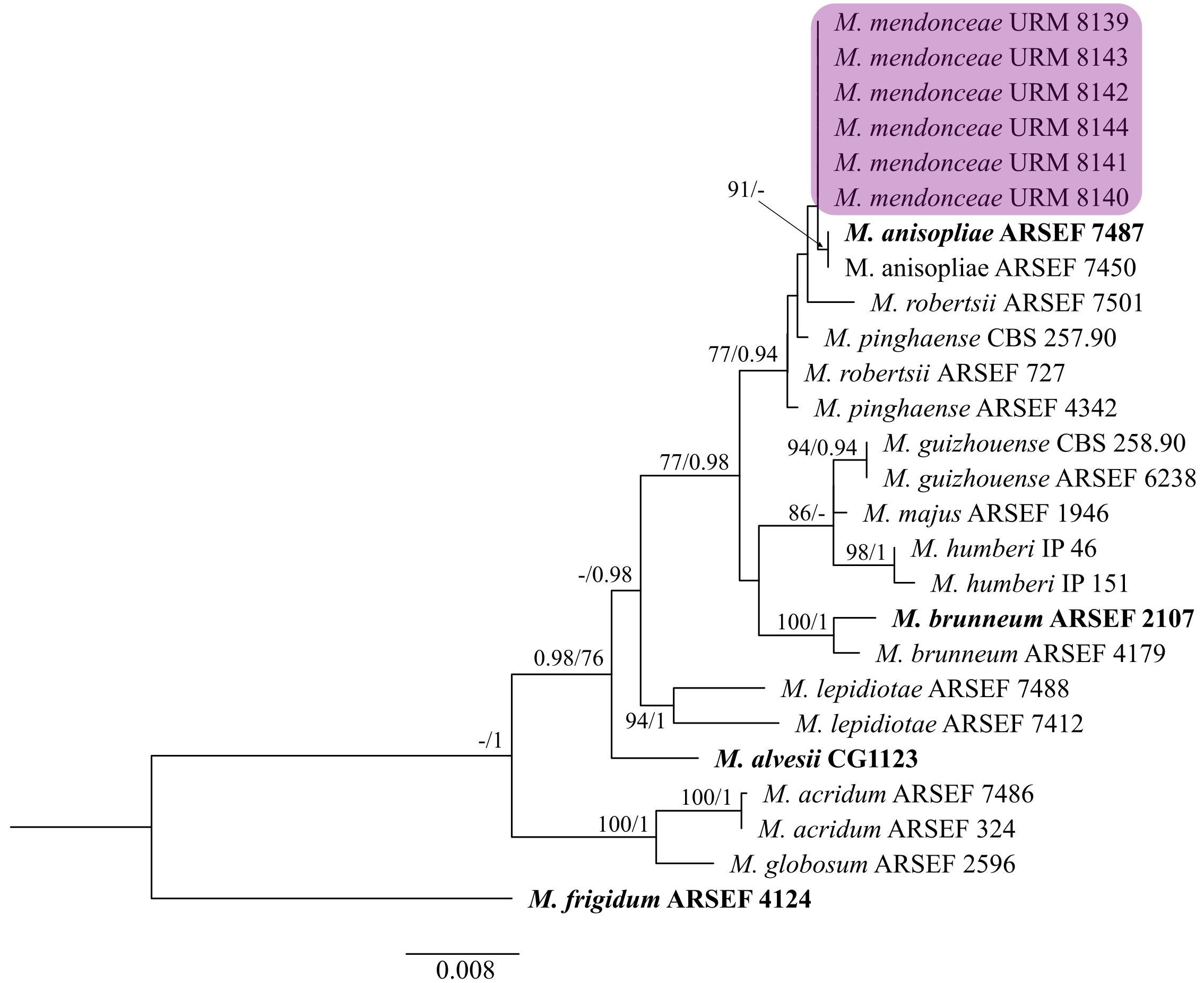

Supplement: S3 Fig — Bayesian phylogenetic tree based on RPB1 sequences showing relationships among species of the Metarhizium anisopliae complex. Isolates from this study from Mahanarva spectabilis, Anastrepha sp., and soil are highlighted in bold. Bayesian posterior probability (PP) values > 0.7 are indicated above the nodes. The symbol T refers to ex-type isolates. This tree is rooted in M. fluorescens. (JPG) [file pone.0310548.s003.jpg]

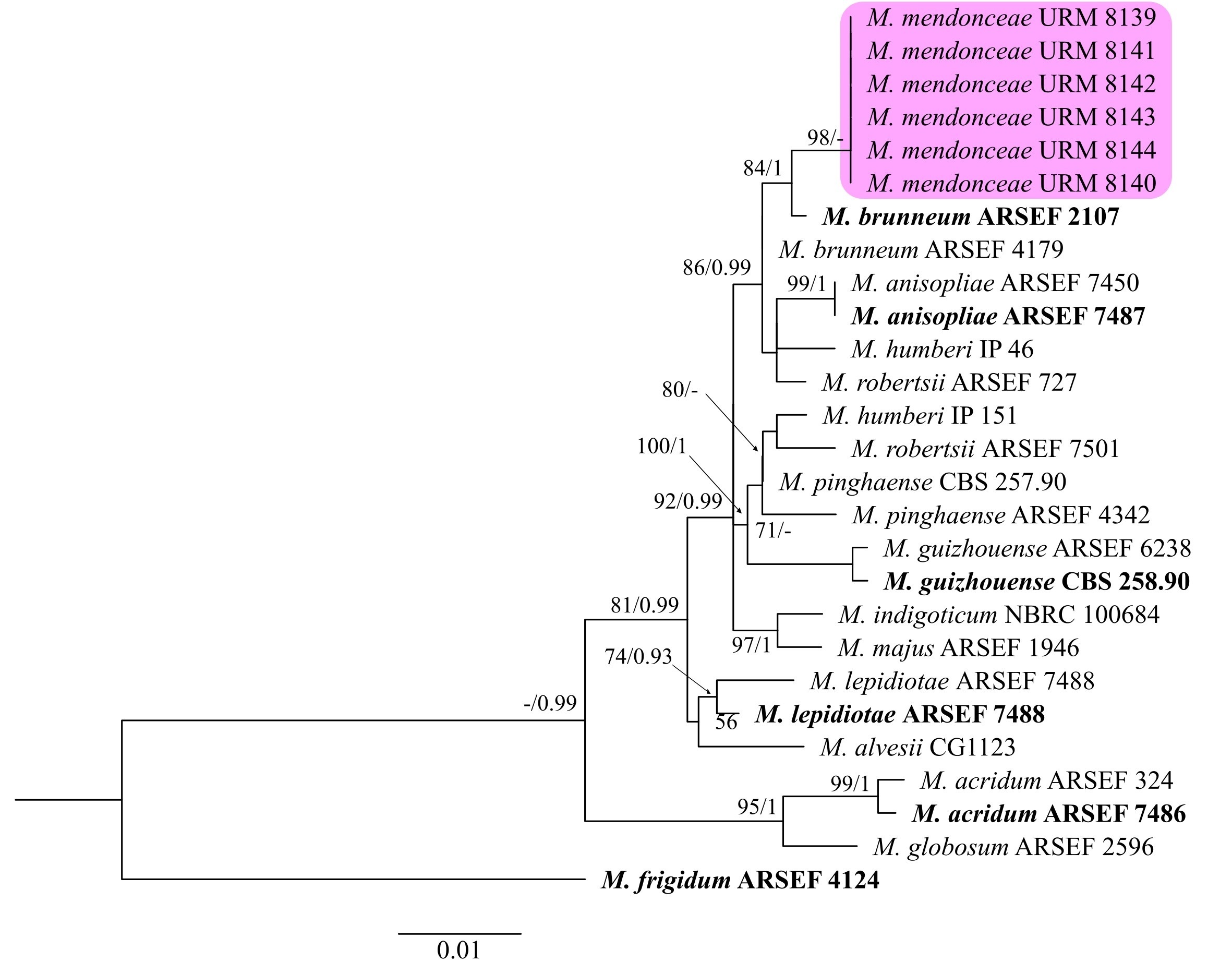

Supplement: S4 Fig — Bayesian phylogenetic tree based on RPB2 sequences showing relationships among species of the Metarhizium anisopliae complex. Isolates from this study from Mahanarva spectabilis, Anastrepha sp., and soil are highlighted in bold. Bayesian posterior probability (PP) values > 0.7 are indicated above the nodes. The symbol T refers to ex-type isolates. This tree is rooted in M. fluorescens. (JPG) [file pone.0310548.s004.jpg]
